# Unusual Placement of a Central Venous Catheter: Left Pericardiophrenic Vein

**DOI:** 10.5811/westjem.2015.2.25656

**Published:** 2015-04-02

**Authors:** Karim El-Kersh, Rodrigo Cavallazzi, Mohamed Saad, Juan Guardiola

**Affiliations:** University of Louisville, Department of Pulmonary, Critical Care, and Sleep Disorders Medicine, Louisville, Kentucky

A 62-year-old man presented to the emergency department with hypotension and diarrhea secondary to Clostridium difficile infection. Due to poor peripheral access, a left internal jugular vein triple lumen central venous catheter (CVC) was inserted for fluid resuscitation. The CVC was placed under real-time ultrasound guidance, which revealed normal anatomy, with no resistance during placement. Good blood return was noted in all three ports. Follow-up chest radiograph showed an abnormal course of the CVC (Figure 1). Despite the abnormal course, blood gas analysis and pressure transduction via the CVC were consistent with venous placement. Chest computed tomography without contrast revealed placement of the CVC in the left pericardiophrenic vein (Figure 2).

Left paramediastinal central line position can be extravascular with direct placement in the mediastinum or pleural space, arterial with extension into the descending thoracic aorta, or venous. Differential diagnosis of venous left paramediastinal CVC position includes left-sided superior vena cava, left internal mammary vein, left superior intercostal vein and left pericardiophrenic vein.^1^ The left pericardiophrenic vein accompanies the left pericardiophrenic artery and the left phrenic nerve along the left pericardium before joining the floor of the left brachiocephalic vein opposite to the entrance of left internal jugular vein. Misplaced catheter tip can migrate into the pericardial space resulting in cardiac tamponade due to fluid administration into the pericardium.^2^ The use of central venous catheters should be postponed, if possible, until a chest radiograph has documented correct placement.

## Figures and Tables

**Figure 1:**
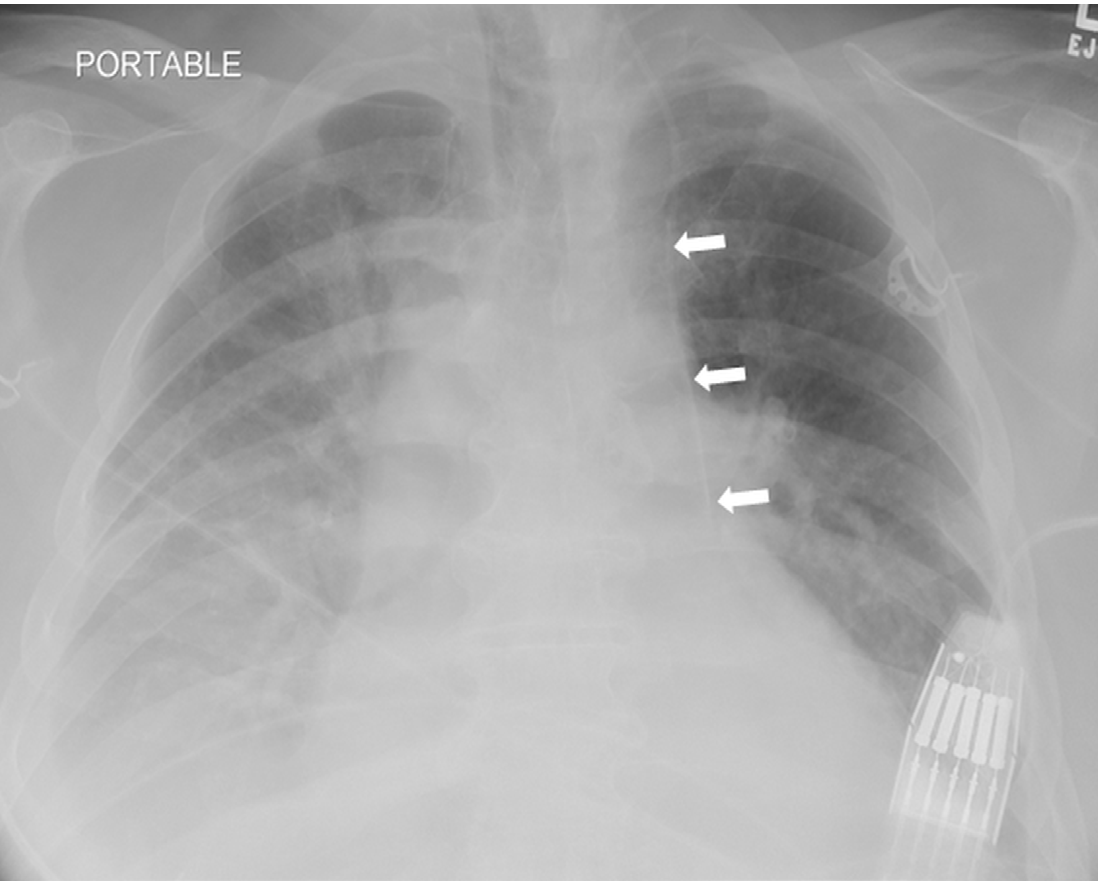
Chest radiograph shows left paramediastinal position of the central venous catheter (arrows) inserted via left internal jugular vein.

**Figure 2:**
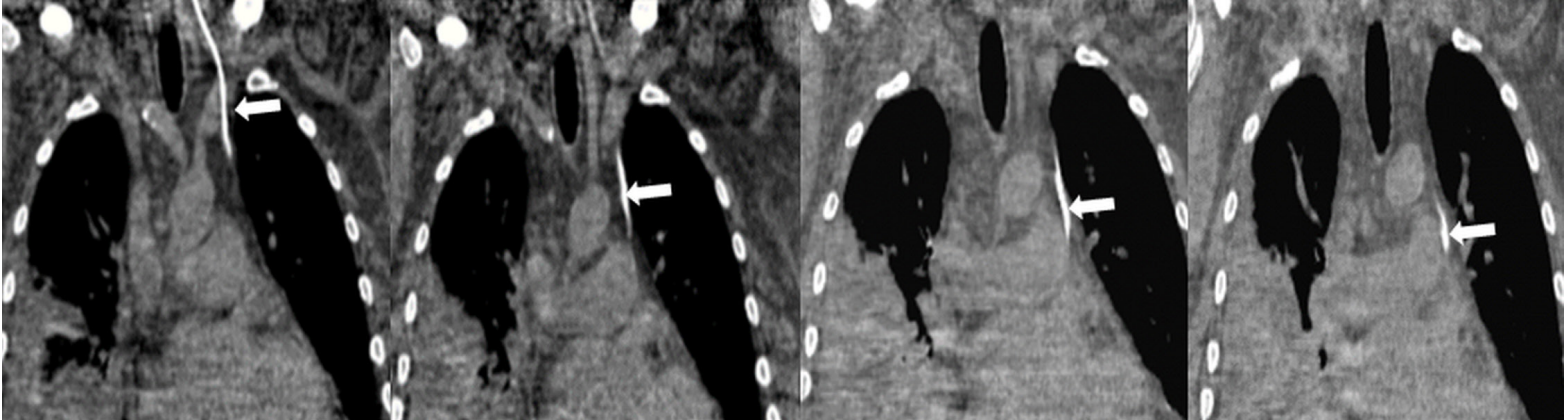
Chest computed tomography without contrast (coronal sections) shows the course of the CVC (arrows) descending via the left internal jugular vein, crossing the left brachiocephalic vein, and then descending through the left pericardiophrenic vein. *CVC*, central venous catheter
